# How practically feasible is tailored screening for cervical cancer based on human papillomavirus vaccination status?: the role of data linkage and registers

**DOI:** 10.1093/jncimonographs/lgag014

**Published:** 2026-08-31

**Authors:** Partha Basu, Eric Lucas, K Miriam Elfström

**Affiliations:** Early Detection, Prevention and Infections Branch, International Agency for Research on Cancer, Lyon, France; Early Detection, Prevention and Infections Branch, International Agency for Research on Cancer, Lyon, France; Center for Cervical Cancer Elimination, Karolinska Institutet and Karolinska University Hospital, Stockholm, Sweden

## Abstract

There is substantial evidence examining effectiveness of human papillomavirus vaccines in populations using registry linkages. These studies initially mapped early outcomes, but in recent years have focused on harder endpoints, confirming that the vaccines have the anticipated effect against infection, lesions, and ultimately invasive cervical cancer. It is generally accepted that when vaccinated cohorts enter screening programs, adjustments to screening strategies are needed to maintain the balance of benefits and harms and achieve optimal resource use. The purpose of this chapter is to examine the practical considerations for implementing tailored screening in the context of increasingly vaccinated populations, with particular emphasis on data sources and their application in routine practice. We present real-world examples of linking vaccination and screening registries to enable screening strategies informed by individual human papillomavirus vaccination status. We also outline the common challenges encountered when establishing such linkages and offer practical suggestions for overcoming them.

## Introduction

Implementation of vaccination against human papillomavirus (HPV), combined with population-based screening using validated HPV detection tests, has the potential to eliminate cervical cancer when both programs achieve high coverage.^1^ The global landscape of cervical cancer prevention is expected to evolve rapidly as cohorts of vaccinated women reach screening age. These women have a substantially lower risk of high-grade cervical precancers (cervical intraepithelial neoplasia grade 2 or worse [CIN2+]) and cervical cancer and therefore would require much less intensive screening (described in detail later in this article). Furthermore, vaccination changes the type-specific prevalence in lesions, and given different oncogenicity across types, follow-up strategies in vaccinated populations will also have to be adjusted. To improve efficiency, screening initiation can be delayed, intervals can be extended, and women with consecutive negative HPV tests may not need further screening after 2 or 3 rounds.

Risk-based screening applies not only to women with documented vaccination status but also to women living in highly vaccinated populations. High coverage of girls (and of boys in gender-neutral vaccination programs) generates herd immunity that provides significant protection even to unvaccinated women in the same birth cohorts.^2^ Risk-based screening is increasingly considered, because less intensive screening reduces program costs and improves the balance of benefits to harms by avoiding unnecessary investigations, treatments, and associated side effects. However, its implementation depends heavily on data quality, legal and governance frameworks, information technology (IT) systems, and the overall health system context.^3^

A critical requirement for risk-based screening is the availability of robust information systems documenting HPV vaccination (vaccination registries) and screening-related activities from invitation to follow-up (screening registries), with the capability to link data reliably at the individual level. A cancer screening registry is defined as a specialized, systematic information system that collects and manages data on individuals participating across the entire continuum of services (invitation, administration of screening tests, diagnostic evaluation, management, and follow-up).^4^ A vaccination registry documenting the type and doses of vaccines received by each recipient with capacity to recall for missed dosages is relatively simpler.

Linkage between screening and vaccination registries not only enables implementation of risk-based screening with periodic review of strategies but also is essential for monitoring vaccine effectiveness in reducing HPV prevalence and cervical precancers and cancers in the population. Linking either the vaccination or screening registry with population-based cancer registries is necessary to measure the ultimate impact of HPV vaccination and screening, a reduction in cervical cancer incidence and mortality. Such registries also support the programs to achieve high coverage and quality through regular measurement and monitoring of key performance indicators.

With most HPV vaccination programs now transitioning to a single-dose schedule for adolescent girls, it is essential to closely monitor the long-term impact of this reduced-dose strategy. Robust linkage between the registries will enable accurate assessment of the durability of protection conferred by a single dose, an issue of major public health importance. Such surveillance systems are critical to ensuring sustained confidence in HPV vaccination programs and guiding future immunization policies.

Our present review article highlights the programmatic benefits of vaccination and screening registries especially in the context of offering tailored screening, the key implementation challenges, and possible solutions.

## Programmatic context for establishing HPV vaccination and screening registries

Linkage between vaccination and screening registries and population-based cancer registry is a relatively new concept even in higher resourced settings. Though such linkages were initially established in some countries purely to monitor HPV vaccination impact, linkage is increasingly being appreciated as screening recommendations are redrafted to incorporate strategies based on individual- and population-level risks. In the following section we briefly describe the role of vaccination and screening registries in the broader context of programmatic implementation and monitoring.

### HPV vaccination registry

A vaccination registry’s primary functions are to improve coverage monitoring, support reminder and recall systems, and strengthen surveillance of immunization programs. An HPV vaccination registry, when linked with screening and cancer registries, becomes an especially valuable resource. Such linkage allows countries to monitor the long-term effectiveness of a unique vaccine for which several decades typically elapse between the age of primary vaccination and the age at which the targeted disease commonly arises.

Following licensure and market availability of HPV vaccines, different study designs have been used to measure the effect of vaccination on HPV-associated disease outcomes. Early studies examined ecological trends focusing on reductions in condyloma and prevalence of HPV before and after availability of vaccines.^5-7^ As more follow-up time became available, effectiveness analyses were possible on an individual level, linking health-care registries to vaccination registries to compare vaccinated with unvaccinated individuals. Studies have looked at reduction in incidence and prevalence of condyloma, precancerous lesions (CIN 2+), and more recently, linkages to cancer registries have been made to examine effectiveness of vaccination against the hardest endpoint—invasive cervical cancer.^8-10^ These analyses have been done in the research setting and contributed significantly to our understanding of the impact of vaccination in the population, and have allowed us to better describe the effect of dosing schemes, age at vaccination, and herd protection.

Strategies for delivering HPV vaccines differ across countries, and therefore registration systems for administered doses also vary. Over time, strategies have shifted within countries as well meaning that registration of administered doses may be captured in different sources as programs extend their reach or become more systematic. As an example, first-generation vaccines came onto the market in Sweden in 2006 and 2007, and school-based vaccination was nationally implemented in 2012. In the years between, administered doses with consent were registered individually in the Swedish HPV Vaccination Register. Since inclusion of HPV vaccination in the childhood vaccination program, doses have been registered in the National Vaccination Register. In the early years, doses were also captured in the Prescription Drug Registry.^11^ Using these sources, the coverage of vaccination can be mapped by geography, birth cohort, and sex. Researchers with ethical approval can link these data to other health registries to map vaccination reach and impact. However, linkages between registries in routine health care are not possible by regulation in Sweden and some European countries. Individual-level vaccination status therefore cannot be linked to the screening call-recall systems to determine individual pathways for vaccinated individuals. That said, these data provide reliable monitoring systems that can be used to inform strategies in conjunction with knowledge of timing of program extension to implement tailored screening strategies.

Several low- and middle-income countries (LMICs) have leveraged integrated digital health information systems to register the HPV vaccine recipients. In Bangladesh, the girls are registered with a software (https://vaxepi.gov.bd) common for all vaccinations.^12^ Registration is done using a birth registration number that helps confirm age eligibility of the recipients. Human papillomavirus vaccination was launched in India in February 2026 with mandatory registration of girls aged 14 years on Universal Immunisation Programme-WIN mobile or web platform.^13^ Once registered, a parent or guardian can access and sign the consent form, receives appointment reminders, and receives a digital vaccination record postvaccination. The backend databases of such applications or online platforms act as vaccine registries, allowing the programs to keep track of coverage by birth cohorts and by regions.

### Screening registry

Organized, population-based cervical cancer screening makes use of call-recall systems to manage invitations and facilitate recall for evaluation of test-positive results and re-invitation for subsequent screening episodes. Screening registry is a centralized data system to support these activities with information on the target population, outcomes of performed tests, further assessments, and final diagnoses, communicating with laboratory information systems, hospital medical records, and population registries. Systematic data extracts from laboratory information systems are preferable to avoid individual data entry but must be standardized to ensure concordance over different systems and mapping of changes over time. Information from medical charts (eg, to capture clinical follow-up of test-positive women) can be more variable and time-consuming to collate, though it is useful for monitoring the entire screening pathway. Systems are adapted to local IT infrastructures and regulations, with an organization of information that matches the local needs.

Depending on the level of organization of the program (county, region, national), when conducting routine monitoring and evaluation of key performance indicators, data are collated and analyzed. Key performance indicators have traditionally been divided into 3 main categories covering screening intensity; screening test performance; and diagnostic assessment, treatment, and posttreatment follow-up. The category of screening test performance includes, among other points, indicators for evaluating the distribution of screened women positive at the primary screening test, detection rates of CIN, and incidence of cancer.^14^ Where possible, indicators should be calculated to capture potential disparities in program coverage, uptake, and follow-up by geography or vulnerability, for example socioeconomic factors, religion, and ethnicity.

In the era of HPV-based screening and impact of vaccination on screening outcomes, these indicators can give important information on the burden of infections and disease outcomes. Where possible, information on HPV type–specific prevalence in screening results gives critical information on when vaccination impacts screening outcomes. A majority of the HPV detection tests validated for screening provide information on at least HPV types 16 and 18, common targets for all HPV vaccines. An effective screening registry would enable trend analyses of these HPV types in the screened population. As an example, the Swedish National Cervical Screening Registry reported a dramatic 98% and 99% decline in HPV 16 and HPV 18 prevalence, respectively, among vaccinated women born in 2000 compared with unvaccinated women born in 1984.^15^ In addition, data collected through a screening registry will help monitor the reduction in the load on colposcopy and treatment services—a significant benefit of vaccination with implication on resource utilization.

**Figure 1.**
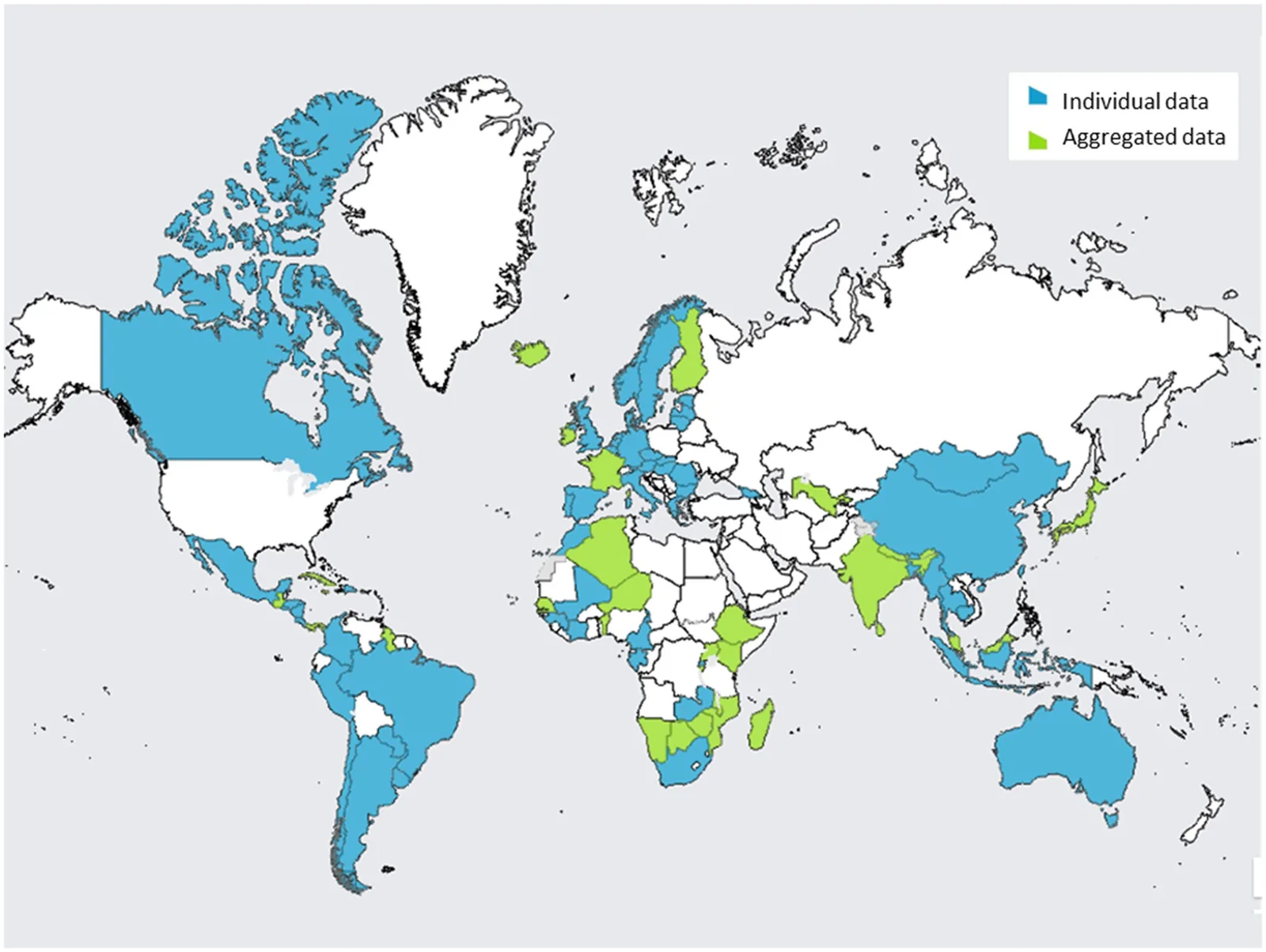
Map showing availability of information system collecting individual-level or aggregate data from cervical cancer screening programs reported in the CanScreen5 project of International Agency for Research on Cancer (report date November 2025).

Organization and systems for data collection in cancer screening programs in LMICs differ markedly from those in higher-resourced settings. Findings from the CanScreen5 project of the International Agency for Research on Cancer (IARC), which compiled information from cervical cancer screening programs in more than 100 countries, highlight this disparity.^16^ Only a small proportion of programs in sub-Saharan Africa or South Asia, regions with some of the highest cervical cancer burdens, have the capacity to collect individual-level data across the full screening pathway (Figure 1). This limited data infrastructure constrains program monitoring, evaluation, and quality improvement and poses challenges for implementing more advanced or risk-based screening strategies.

### Experiences with linkage between screening and vaccination registry

The scope and the technical and regulatory requirements for establishing registry linkages are very much country or program specific. As a typical well-organized screening program in higher resourced settings, the Swedish program is implemented at the regional level according to national recommendations. Each regional program coordinates call-recall activities linking the population registry from the Swedish Tax Agency to laboratory information systems and defined screening algorithms. Data are then extracted directly from information systems in each region and compiled in the National Cervical Screening Registry (www.nkcx.se) for monitoring, evaluation, and program improvement. All invitations, screening test results (primary and triage), and histopathologies are obtained. Key performance indicators are calculated according to recommendations by the National Board of Health and Welfare^17^ and published annually.^18^ As the program has shifted from primary cytology to HPV-based screening and optimized from testing for any HPV positivity (14 types) to extended genotyping, screening data can be used to evaluate HPV prevalence in population. As described earlier, it is feasible to perform analyses of screening data to map HPV prevalence by birth cohorts that have aged into the screening program and were vaccinated in the school-based program.^15^ However, the regulations do not permit linkage for the screening registry to send individualized screening invitation based on vaccination status.

Italy developed a tailored screening strategy for cohorts of women vaccinated at age 12 years, issued by an expert group convened by the National Screening Monitoring Centre of the Ministry of Health and the Italian Group for Cervical Screening.^19^ As part of the National Cancer Prevention Plan, they advised integrating vaccination registries, screening registries, and cancer registries at the regional level, supported by adequate planning and resources. Until full integration of vaccination records is achieved, the experts recommended that screening programs systematically collect self-reported vaccination information from women. This should include whether they were vaccinated, the number of doses received, dates of vaccination, and the type of HPV vaccine administered.

Many of the Latin American countries such as Argentina, Brazil, and Chile use a national online information system for coordination of various health services including cervical cancer screening programs. One such example is the SITAM database (Sistema de Informacion de Tamizaje) deployed nationally but managed at provincial level for capturing cervical cancer screening activities in Argentina.^20^ The primary care facilities, cytology laboratories, and the gynecologists’ services are linked through the system that has registered more than 1.5 million women screened in public institutions. A new module for capturing HPV test results was added as the country switched to HPV detection–based screening in 2015 following the successful implementation of a demonstration project in Jujuy province.^21^ Though individual-level data captured in SITAM allows the program to track and recall HPV-positive women, there is no linkage with the vaccination data. The HPV vaccination program introduced in 2011 captures data in the Nominated Federal Vaccination Registry implemented by the National Programme for the Control of Preventable Diseases. The registry keeps track of HPV vaccines provided in the public and private sectors. However, no study has evaluated Nominated Federal Vaccination Registry data architecture and completeness to register HPV vaccination, as well as barriers regarding data linkage and the use of nominalized data to evaluate impact of the vaccine. A study that reported high effectiveness of HPV vaccination in reducing HPV prevalence in Argentinian women in 2020 did not use the screening or vaccination registry data.^22^

Some of the countries with limited resources customized the open-source software platform DHIS2 with its Tracker function (https://dhis2.org) to collect individual data of the participants to cervical cancer screening. One such example is the screening program in Bangladesh, which is using the DHIS2-based health information system to register women for visual inspection with acetic acid (VIA) screening in more than 90% of the community clinics in the country.^23^ Individuals registered at the community clinics are successfully linked to the screening clinics and colposcopy centers through their unique national identity number. The Directorate General of Health Services managing the screening database also manages the VaxEPI, an integrated digital platform designed to manage HPV vaccination nationwide in 2024.^24^ Having the screening and vaccination databases in the same platform and the use of a common health identifier for each individual create an opportunity to produce linkage, though no such attempt has been made as the HPV vaccination program was launched only in 2023.^12^

## Recommendations and practical feasibility of tailored screening

The pace of change in screening guidelines is rapid, and to accommodate this, processes for guideline development are changing. Living guidelines and guidelines that reflect the changing risk in population will be critical to calibrate the most effective programs, avoiding potential harms of overscreening and its downstream effects. The feasibility of tailored screening is therefore closely related to whether we can effectively capture key risk parameters without overcomplicating or delaying implementation. As discussed earlier, few settings can individually link between vaccination registries and health-care registries. Considering these challenges, recently published guidelines on screening the vaccinated populations in Europe recommend tailored screening based on vaccination coverage of the cohorts of women undergoing screening.

The population-level impact of HPV vaccination depends on vaccination coverage, type of vaccine used, and whether girls-only or gender-neutral vaccination strategy was used and is likely to vary substantially between countries and also between different birth cohorts undergoing vaccination within the same country.^2^ The European Commission Initiative on Cervical Cancer (EC-CvC) led by IARC, in order to update the cervical cancer screening guidelines for European Union Member States, convened a working group of experts to develop recommendations for tailored screening in vaccinated populations.^25^ Given the substantial challenges in obtaining reliable individual-level vaccination data in the absence of effective registry linkage, the group concluded that tailored screening should be based on the vaccination coverage of the cohort entering the screening program rather than on individual vaccination status.

De Bondt et al., in an analysis comparing model predictions with independently published population-based data on the real-world impact of HPV vaccination, demonstrated a clear relationship between cohort vaccination coverage and the relative risk of HPV-related outcomes.^26^ They reported relative risk estimates for HPV16 and 18 prevalence ranging from 0.02 to 0.68 in studies with at least 45% girls-only vaccination coverage, showing a linear trend of increasing protection with increasing coverage. For CIN2+, the relative risk ranged from 0.29 to 0.71 in studies where coverage exceeded 50% for girls-only vaccination.

Drawing on this evidence and a modeling work done by IARC, the EC-CvC working group recommended risk-based screening for cohorts achieving at least 50% vaccination coverage, while cohorts with lower coverage should continue to follow the regular screening strategy applicable to unvaccinated populations. The EC-CvC Working Group suggested start age and screening interval for birth cohorts according to their risk of cervical cancer (defined by vaccination coverage [<50%, 50%-75%, and >75%] and prevaccination HPV prevalence: high [≥6%] and low [<6%]).

Similar approach was taken by the Italian expert group while recommending postponement of screening initiation with HPV test from 25 years to 30 years for the vaccinated cohorts.^16^

Further tailoring of age at screening initiation and screening interval may be feasible based on screening coverage. More than 90% reduction in risk of CIN2+ (including cervical cancer) has been observed in studies reporting 90% vaccine coverage.^26^ However, more complex tailoring requires regular monitoring of coverage and making more challenging strategic adjustments. Screening program data, given that countries have switched to HPV-based screening with some genotyping, gives sufficient surveillance data to adjust strategies to HPV prevalence in the population and monitor further potential shifts in type prevalence. Prevalence obtained in screening gives real-time evidence of decreased risk in the population and allows for tailoring programs, taking into account the direct effects of vaccinated populations as well as herd effects. Overarching vaccination program data can be used to monitor coverage and anticipate when impact in screening outcomes can be expected. As guidelines are updated at an increasing pace to follow advances in our understanding of risk, changes will also be necessary in communication strategies and materials, which require time and focus on messaging. Communication strategies and messages should be locally contextualized and developed for the population targeted for screening and health professionals associated with the programs.

## Practical steps for implementing linkage between vaccination and screening registries

Tailored screening is best achievable with reliable and legally appropriate linkage between registers and clinical data. The key challenges are lack of single-identifier, siloed databases with low quality of data, legal and privacy constraints for data sharing, and large upfront investment in information technology and data governance.

A blueprint needs to be prepared for data infrastructure comprising the system and tools to manage and process data for cervical screening and HPV vaccination programs. Such a data infrastructure would include requirements for hardware, software, networks, and protocols that would describe data input, storage, retrieval, and analysis, ensuring it is secure, accessible, and usable for decision-making. A plan needs to be included for a data warehouse to store and access structured data and a data infrastructure strategy for managing and using data systems effectively. Sustainability and flexibility of monitoring and evaluation are dependent on the accessibility of databases and clarity of data queries such that updates can be made without cumbersome or extensive support.

The blueprint as an operational document should cover the following aspects:

- A data governance framework to describe policies and procedures to ensure that data are accurate, complete, secure, and accessible, while also helping organizations comply with regulatory requirements. The framework will focus on 4 critical pillars:
   ○ Accuracy: ensuring data are as precise and error-free as possible
   ○ Completeness: maintaining full data records, which may involve sourcing additional data
   ○ Timeliness: providing up-to-date data when needed, as outdated data are no better than inaccurate data
   ○ Compliance: ensuring data handling aligns with industry regulations and legal requirements
- Technical requirements to ensure dataflow across various levels of facilities and mechanisms to establish individual-level linkages
- Infrastructural and financial considerations to establish, improve, and sustain the information system
- Training of service providers, data managers, and program managers on managing the information system.
- Operational aspects of the system for managing, monitoring, and quality evaluation of the programs
- Maintaining data quality and consistency
- Pilot testing to check usability, data quality, and interoperability and refining workflows before national roll-out

Aside from complicated logistical and regulatory aspects to implementing individual linkages guiding tailored screening strategies for vaccinated individuals, there are meaningful ethical considerations. Care needs to be given regarding communication around information systems guiding screening programs and individual tailoring of pathways. For example, in Sweden, individuals could opt out of registries early on, which meant that registries may not be fully represented in the underlying parameters that would guide determination of someone’s screening pathway. Alternately, relying on self-report of vaccination status is not reliable and can be problematic if it is associated with more or less access to screening.

## Conclusion

Screening for cervical cancer using HPV testing, combined with various triage strategies, has created multiple programmatic options. As evidence on the risks associated with different combinations of screening test results becomes more detailed, there is a tendency to translate this knowledge into increasingly complex screening algorithms. Tailoring screening based on vaccination status has the potential to increase efficiency, but it also requires sophisticated IT systems that demand substantial time, effort, and resources to develop and maintain. Program optimizations further add to the workload, and delays can occur when guidelines shift to more frequent update cycles.

A more pragmatic approach is to summarize detailed registry data into broader risk levels that guide risk-based strategies. Vaccination registries can be used to identify overarching patterns in coverage—by age, sex, and birth cohort—because these factors influence effective thresholds for reducing HPV prevalence. Screening recommendations can then be adapted for cohorts or age groups with high coverage, building on existing monitoring systems within screening programs. This approach leverages available registry data while allowing flexibility based on the granularity of information accessible in different settings.

In LMICs, programmatic priorities differ. The immediate need is to strengthen the organization of screening services and ensure that at least 70% of women aged 30-49 years are screened twice in their lifetime using validated HPV detection tests, irrespective of vaccination coverage levels. In these settings, robust screening registries are essential primarily for guaranteeing high coverage and ensuring that screen-positive women receive appropriate follow-up and management rather than for implementing risk-based screening strategies.

## Data Availability

The data underlying this article will be shared on reasonable request to the corresponding author.
